# Molecular Interactions of Carbapenem Antibiotics with the Multidrug Efflux Transporter AcrB of *Escherichia coli*

**DOI:** 10.3390/ijms21030860

**Published:** 2020-01-29

**Authors:** Alessio Atzori, Giuliano Malloci, Francesca Cardamone, Andrea Bosin, Attilio Vittorio Vargiu, Paolo Ruggerone

**Affiliations:** Department of Physics, University of Cagliari, 09042 Monserrato (CA), Italy; alessio.atzori@dsf.unica.it (A.A.); francesca.cardamone@dsf.unica.it (F.C.); andrea.bosin@dsf.unica.it (A.B.); paolo.ruggerone@dsf.unica.it (P.R.)

**Keywords:** antibiotic resistance, Gram-negative bacteria, resistance nodulation-cell division transporters, AcrB, molecular docking, molecular dynamics simulations, binding free energy calculations

## Abstract

The drug/proton antiporter AcrB, engine of the major efflux pump AcrAB(Z)-TolC of *Escherichia coli* and other bacteria, is characterized by its impressive ability to transport chemically diverse compounds, conferring a multi-drug resistance (MDR) phenotype. Although hundreds of small molecules are known to be AcrB substrates, only a few co-crystal structures are available to date. Computational methods have been therefore intensively employed to provide structural and dynamical fingerprints related to transport and inhibition of AcrB. In this work, we performed a systematic computational investigation to study the interaction between representative carbapenem antibiotics and AcrB. We focused on the interaction of carbapenems with the so-called distal pocket, a region known for its importance in binding inhibitors and substrates of AcrB. Our findings reveal how the different physico-chemical nature of these antibiotics is reflected on their binding preference for AcrB. The molecular-level information provided here could help design new antibiotics less susceptible to the efflux mechanism.

## 1. Introduction

**Figure 1:**
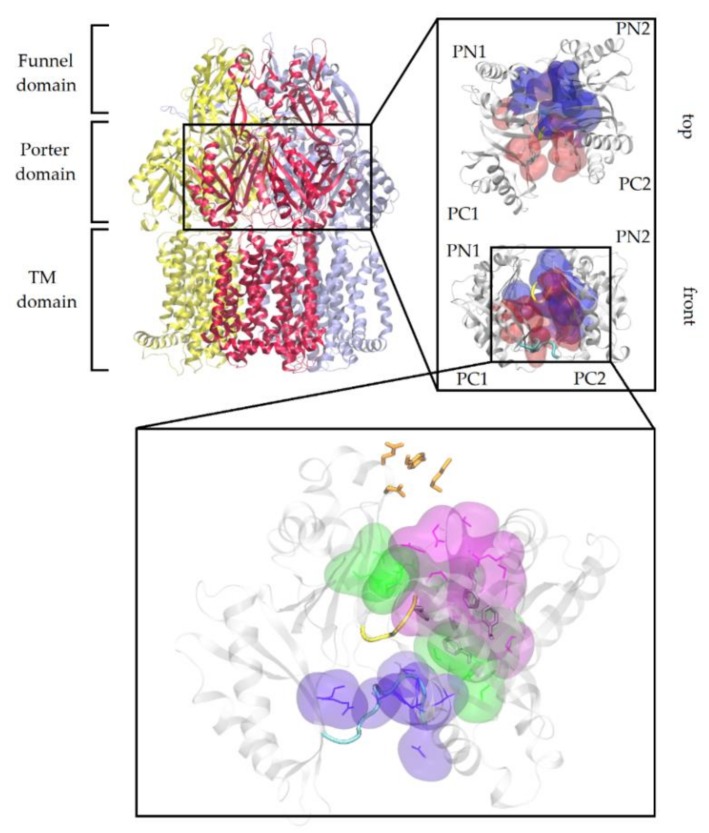
View of the AcrB trimer. The access, binding, and extrusion protomers of AcrB are represented as yellow, red, and ice blue ribbons, respectively. The inset shows a front and top magnification of the porter domain of the T monomer of AcrB highlighting PN1, PN2, PC1, and PC2 subdomains and the access and deep binding pockets represented as red and blue surfaces, respectively. The second inset shows the main regions of interest discussed in this study, as reported in Table S1. The switch-loop and the bottom loop are displayed as yellow and cyan tubes, respectively. HP-trap and exit gate residues are shown as gray and orange sticks, respectively. Interface, groove, and cave residues are represented as violet, green, and purple surfaces and lines respectively. Protein residues lining each region are reported in Table S1.

The increased number of MDR Gram-negative bacterial strains, along with the reduced efficacy of available antibiotics, constitute a significant concern for public health [1,2,3]. A prominent role in the MDR phenotype is played by resistance nodulation-cell division (RND) efflux pumps [4,5,6,7,8,9,10,11,12], complex poly-specific transporters involved in the extrusion of a wide range of chemically unrelated compounds. The inner membrane drug/proton antiporter AcrB, part of the major AcrAB(Z)-TolC efflux system of *Escherichia coli,* is one of the most investigated members of the RND superfamily [6,13,14,15,16,17,18,19]. Structurally, AcrB is a homotrimer and each of its protomers is composed of a transmembrane (TM) domain (where a proton gradient provides the energy needed for substrate translocation [20]), a porter domain (responsible for the recognition, uptake, and first transport of substrates), and a funnel domain (constituted by a channel linked to partner proteins AcrA and TolC) (Figure 1).

Both a symmetric and an asymmetric conformation of AcrB, thought to represent its resting and active states respectively, have been identified by X-ray crystallography [13,14,16,21,22,23,24]. In the asymmetric structure, each protomer is found in a different conformational state defined as loose (L), tight (T), and open (O) [13,18,19]. This finding suggested that the efflux of substrates is induced by a functional rotation mechanism [13,14,15], where each protomer can alternatively assume the above conformations in concert with the others. This hypothesis has been supported by subsequent experimental [25,26] and computational studies [27,28,29,30,31]. Additionally, the available asymmetric structures of AcrB allowed for the identification of specific substrate recognition sites: (i) the access pocket (AP), located in the vestibule region between PC1 and PC2 subdomains and open in protomers L and T [16,32]; (ii) the distal pocket [16,32], located more closely to the funnel domain and open only in the T protomer (hereafter DP_T_; see Table S1 for the list of residues belonging to different regions of AcrB). Recently, a new binding site localized in the transmembrane region has been identified [26].

Due to its crucial position in the entire efflux pathway, the DP_T_ is supposed to interact with all AcrB substrates along their transport pathway, independently of their molecular size and physico-chemical features. This hypothesis has been recently supported by the publication of the co-crystal structures of high molecular mass compounds bound within the DP_T_ of MexB, the homologous protein of AcrB in *Pseudomonas aeruginosa* [33]. The portion of the DP_T_ comprising multiple phenylalanine residues (136, 628, 610, 615, 628) and named “hydrophobic trap” (HP-trap) [34], is a crucial recognition site for AcrB inhibitors [35,36], and was also shown to interact with several substrates [16,32,37,38,39]. The interface between access and distal pockets is constituted by a glycine rich loop (aka “switch loop”), which regulates the transition of substrates towards the DP_T_ [16,32,40]. A “bottom-loop”, which could play a role as well in the recognition and transport of substrates, is found oppositely to the switch-loop within the access pocket [40,41,42] (Figure 1).

A detailed understanding of the relationship between physico-chemical properties of antibiotics and their propensity to be expelled by efflux pumps is relevant to understand the mechanisms of polyspecific transport and can be very informative for drug design campaigns. Due to the complexity of RND transporters and the difficulty of producing co-crystals, only a few X-ray structures of the asymmetric trimer of AcrB bound to substrates have been reported to date [13,14,15,16,18,20,26,32,34]. The characteristics of compounds co-crystallized so far led to the conclusion that a certain degree of hydrophobicity is required for substrates of AcrB [43]. Computational studies can provide atomistic information on the dynamics of biomolecular complexes. As such, they represent a viable alternative to gain insights into the binding of compounds to AcrB. One of the earlier studies on this protein identified two distinct sub portions of the DP_T_, called “groove” and “cave” [44,45]. The groove region is located in the upper portion of the DP_T_, close to the so-called exit gate (EG, Table S1) [15], while the broader cave region is located at the bottom of the DP_T_. Later, molecular dynamics (MD) simulations have been used to investigate substrate binding and specificity of AcrB [37,38,46], as well as to unveil the link between structural-chemical fingerprints of compounds and physico-chemical properties of the multidrug binding sites of AcrB [42,47]. These studies revealed that compounds can preferentially bind to the cave, to the groove or to both regions, but no clear rules were identified explaining the binding preferences of different compounds. Indeed, almost iso-energetic binding modes were predicted for the same ligand, a result compatible with the so-called oscillation hypothesis [19], according to which AcrB substrates oscillate between different binding modes with similar affinity within the DP_T_.

Among the different families of in-use antibiotics, carbapenems represent a primary resource for the treatment of severe bacterial infections [48,49]. Unfortunately, several carbapenem-resistant Gram-negative strains emerged over the years, for which there is evidence that these antibiotics can be recognized and extruded by RND efflux pumps [50,51,52]. These conclusions usually relied on the comparison between minimum inhibitory concentration (MIC) measurements obtained for wild-type and efflux-pumps deficient strains. However, MIC values are strongly influenced by the interaction of the drug with many other bacterial components: for instance, the membrane permeability, heavily dependent on intrinsic drug properties, can definitely surpass the specific contribution of active efflux to MDR [53,54]. As an example, while meropenem is a good substrate of the MexAB-OprM efflux pump from *P. aeruginosa* and imipenem resistance to this organism appears to be predominantly caused by changes in outer membrane pores, the impact of AcrAB-TolC on the activity of IMI and MER in *E. coli* is still under debate [55]. In this context, direct efflux and affinity measurements [56,57,58], combined with a molecular picture of the interaction between these compounds and AcrB, would be pivotal for the design of more effective antibiotics starting from validated and widely used scaffolds.

**Figure 2:**
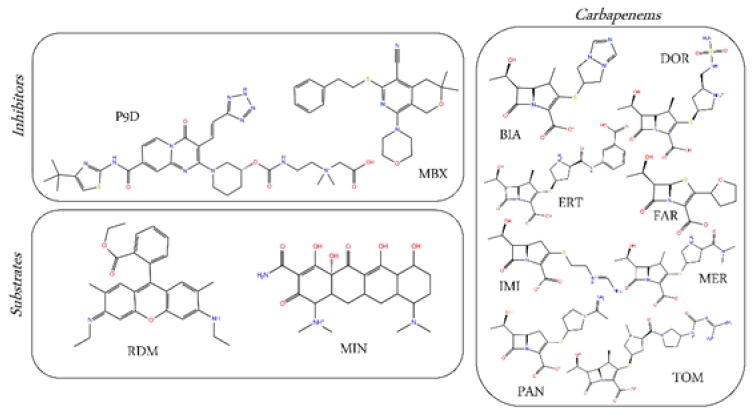
Two-dimensional (2-D) structures of the substrates/inhibitors co-crystallized with AcrB and of the selected carbapenems antibiotics considered in this study.

Along these lines, we report here a comparative computational investigation of the interactions between selected carbapenem antibiotics, such as biapenem (BIA), doripenem (DOR), ertapenem (ERT), faropenem (FAR), imipenem (IMI), meropenem (MER), panipenem (PAN), and tomopenem (TOM) (Figure 2), and the DP_T_ of AcrB. Half of the selected carbapenems (namely DOR, ERT, IMI, and MER) are currently in clinical use. As a reference, two efflux pump inhibitors (MBX3132 and D13-9001, hereafter MBX and P9D respectively) and two known substrates of AcrB (rhodamine-6G and minocycline, hereafter RDM and MIN) was investigated with the same computational protocol adopted for carbapenems (Figure 2).

The outcomes of this work led to the identification of binding modes and specific interactions between chemically related molecules and different sub-pockets within the DP_T_ of AcrB. This study contributes to explain the detailed interactions established by different carbapenem antibiotics with the DP_T_ of AcrB in terms of their intrinsic physico-chemical properties. A retrospective analysis of the previous literature on other antimicrobial compounds [44] revealed the general applicability of our findings [35,55,59,60,61].

## 2. Results

In continuation of our previous studies [37,55,59,60], we combined molecular docking, MD simulations, binding free-energy and hydrophobic/hydrophilic surface-matching calculations, and solvation analyses. Namely, for each compound, one single mode of binding within the DP_T_ of AcrB was found from blind docking calculations. These docking poses (and the crystallographic configurations considered) were used as starting conformations for 100 ns-long all-atom MD simulations in water solution followed by a cluster analysis of the trajectory. Binding free-energy calculations were thus performed on the conformations extracted from the most populated conformational cluster. From the cluster representative configuration, the hydrophobic (SM_L_) and hydrophilic (SM_H_) matching coefficients between the interaction surfaces of the compound and the DP_T_ of AcrB were computed: both quantities range from (no matching) to 1 (full matching). Additionally, the de-solvation of each compound upon binding to AcrB was also estimated from MD simulations (see Materials and Methods for details).

In the following paragraphs, we first present the results for substrates and inhibitors co-crystallized with AcrB; next, we discuss the data obtained for the carbapenem antibiotics considered in this work. The starting X-ray crystal configurations and the selected docking poses as well as the structural cluster representative of each MD simulation can be visualized online at the web address www.dsf.unica.it/translocation/docking. To rationalize our results in terms of specific physico-chemical features of the compounds, we report some molecular properties in Table S2. Molecular weights (MWs) are approximately in the range 300–500 Da, while the total charge ranges from -1 to +1 including several neutral zwitterionic species. Based on available MICs data (compiled in Table S3), the AcrAB-TolC efflux pump would recognize and expel the considered compounds. However, as specified in the Introduction, a detailed atomistic description of the interactions of different compounds within the extrusion channel of AcrB cannot be achieved with current experimental techniques.

### 2.1. Substrates and Inhibitors Co-Crystallized with AcrB

Comparative data available for the crystallographic poses of substrates and inhibitors bound to the DP_T_ of AcrB are summarized in Table 1. Two substrates MIN (PDB ID: 4DX5 [32]) and RDM (PDB ID: 5ENS [36]), and the two inhibitors P9D (PDB ID: 3W9H [34]) and MBX (PDB ID: 5ENQ [36]) were considered. MD simulations revealed overall stability of the crystallographic poses (Figure S1), as further confirmed by the cluster analysis of the trajectory which predicted one single conformational cluster in all cases. The root-mean-square displacement (RMSD) of the cluster representative from the starting X-ray configuration has the value of 2.1, 4.0, 1.7, and 2.4 for MIN, RDM, MBX, and P9D, respectively (Figure S2). The hydrophobic surface-matching analysis showed that the lipophilic surface matching (SM_L_) plays an important role in the interaction observed for all the compounds (values between 0.80 and 0.96). On the contrary, the hydrophilic surface matching (SM_H_) is found to vary from 0.01 (RDM) to 0.91 (P9D) according to the very different chemical nature of these compounds as quantified by the octanol/water partition coefficient logP (Table S2).

No clear trend was found between the dehydration of compounds and their classification as substrates or inhibitors of AcrB (Table 1). Given the highly hydrophobic nature of the DP_T_, a consistent fraction of waters was lost in all cases (from 60% to 70% compared to the value in bulk water).

**Figure 3:**
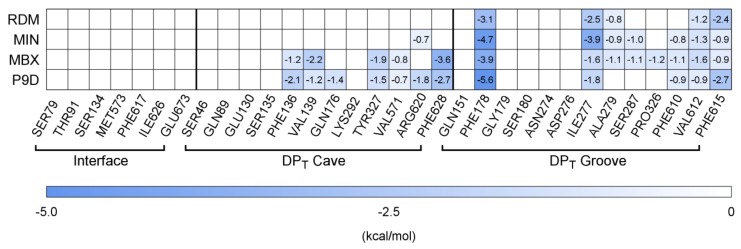
Per-residue contributions to the free-energy of binding (kcal/mol) evaluated for the considered substrates and inhibitors co-crystallized with AcrB. Only residues contributing more than kT (~0.6 kcal/mol at 310 K) are reported. Residues of the interface, DP_T_ cave and DP_T_ groove are colored with different tones of blue according to their individual contribution to the overall ΔG_b_.

As to the strength of the interaction, the inhibitors show higher affinities (larger negative values of the binding free energy ΔG_b_) in comparison to substrates. In order to investigate more deeply the reason for this difference, we analysed the per-residue contributions to ΔG_b_ focusing on residues lining three well-defined portions of the pocket so as to locate spatially the binding mode of each compound: the entrance of the DP_T_ (or the Interface between access pocket and DP_T_), and the cave and groove regions defined in [44] (Table S1). The contribution to ΔG_b_ of the different regions of the DP_T_ reported in Table 1 reveal that: a) the interface turns out to contribute almost nothing (1–2%) to the stabilization of both substrates and inhibitors; b) MIN and RDM appear to be prevalently groove binders (contribution in the range 30–50%); c) MBX and P9D bind the entire pocket, interacting with both the cave and the groove regions (energetic contribution of about 20% for both cave and groove). The detailed pattern observed (Figure 3) reveals the prominent role played by PHE178, ILE277, VAL612, and PHE615 (groove) in stabilizing the binding of all compounds, as well as the contribution of the hydrophobic residues PHE136, VAL139, TYR327, VAL571, and PHE628 (cave) only to the stabilization of inhibitors. Importantly, not only hydrophobic residues (especially the phenylalanines of the HP-trap), but also polar (ARG620) and weakly polar (SER287) residues are found to be relevant. All of the above findings are consistent with previous investigations [35,37] and available structural and biophysical data, validating the adopted computational protocol for a systematic investigation of the binding of carbapenems to the DP_T_ of AcrB.

### 2.2. Carbapenems

Given the absence of experimental data concerning the molecular interaction of carbapenems with the DP_T_ of AcrB, the same computational investigation described for known inhibitors and substrates of this transporter was performed for selected antibiotics of this family.

Table 2 summarizes the results obtained for the carbapenems, sorted by increasing MW. MD simulations revealed that few compounds remained close to the starting configuration (MER, PAN, DOR, see Figure S3), while consistent mobility was observed for the remaining compounds, with RMSDs ranging from 4–5 Å (FAR, BIA, ERT) up to 5–6 Å (IMI, TOM). Contrary to what has been observed for known substrates and inhibitors of AcrB, in the case of carbapenems the hydrophilic surface matching seems to be more relevant than the lipophilic matching (on average, we found higher values of SM_H_ as compared to SM_L_, and in 3 out of 8 cases the latter value almost vanishes). Measured dehydration for carbapenems is similar to that observed for substrates and inhibitors only for FAR, IMI and MER (60–70% of water molecules lost). The other members of the class show less pronounced dehydration in the range 50–60%. The values of ΔG_b_ computed for carbapenems are of the same order of magnitude as that of MIN (about -30 kcal/mol). Notably, in agreement with the results of our previous study [55], MER displays a moderately higher affinity than IMI for the DP_T_ of AcrB (−31 vs. −25 kcal/mol), coupled with reduced mobility. Interestingly, some qualitative trends appear when considering the different contributions to the total ΔG_b_ of the interface, the cave, and the groove regions of the DP_T_. More specifically, the contribution of the interface, which was almost null for known substrates and inhibitors, remains relatively small but roughly anti-correlated to the MW, ranging from ~10% for the smallest compound to zero for the largest one. Except for DOR, MER, and TOM, the contribution of the cave falls in the range 20–30%, on average exceeding the values registered for inhibitors (~20%). On the contrary, while for smaller carbapenems the contribution of groove residues is reduced (~5%), for bulkier compounds we observe the same order of magnitude found for substrates and inhibitors (~30%). The only exception to the above trend is represented by TOM, whose cave and groove contributions are similar and relatively small (~15%).

The above analysis is complemented by the detailed per-residue contributions reported inFigure 4, which reveal a thermodynamic pattern overall different from that shown in Figure 3 for known substrates and inhibitors of AcrB. Firstly, for a few low-MW carbapenems a non-negligible contribution to ∆G_b_ comes from some residues of the Interface region (PHE617 belonging to the switch-loop between the AP and the DP, or THR91, MET573, and ILE626). Secondly, stabilization of low-MW carbapenems appears to be due mostly to residues from the cave, including several hydrophobic but also polar and charged sidechains. Thirdly, at increasing MW, the compounds appear to be more and more buried within the whole DP_T_, stabilized by residues from both cave and groove residues (similar to what is observed for some inhibitors [34,36]; see also Figure S4). Overall, PHE617 of the Interface, PHE136 and ARG620 of the cave, and PHE178, ILE277, and PHE615 of the groove contribute mostly to the free energy of binding of the carbapenems considered in this work.

As expected, consistently with the mainly lipophilic nature of the DP_T_, hydrophobic residues are found to be the majority. Additional residues contributing to the stabilization of some carbapenems, such as GLN89, SER135, TYR327, and ASN274, are known to play a key role in the efflux of substrates mediated by AcrB [62].

## 3. Discussion

Despite the recognized role of RND efflux systems in multi-drug resistance to antibiotics, a quantitative assessment of their contribution remains challenging due to the difficulties in the determination of the efflux kinetics of most substrates of AcrB and homologous RND transporters [58,63,64]. While for other drugs the binding to AcrB has been characterized structurally [32,34,36] and/or by means of site-directed mutagenesis [65,66,67] to the best of our knowledge for the carbapenems investigated in this study, the available data possibly related to efflux-mediated resistance are the variations in the MIC values of antibiotics upon deletion of the outer membrane transporter TolC and/or AcrB (or homologous proteins see e.g., [68,69,70]). These data, however, notoriously reflect additional processes involving antibiotics and other bacterial components [53].

Furthermore, the challenges of obtaining experimental structures of RND transporters in complex with their substrates must be taken into account in this scenario [8]. Henceforth, several computational labs made efforts at determining the molecular interactions between these multi-drug proteins and different compounds, including antibiotics and inhibitors [37,42,44,47,71,72] (for a recent review, see [73] and references therein). The work presented here can be framed in this context and was motivated by the purpose of providing information about the possible interaction between selected carbapenems and AcrB of *E. coli*. Indeed, to the best of our knowledge, no direct efflux measurements are available for this class of compounds. In contrast, whereas kinetic efflux data are available for other families of β-lactam antibiotics [58,63].

The binding propensity of carbapenems to the DP_T_ of AcrB as well as their interaction patterns revealed several remarkable features. First, along with the MD simulations, all compounds but imipenem adopted a relatively stable (along the timescale of tens of ns) binding position within the DP_T_ of AcrB, regardless of the displacement from the initial docking pose (Figures S3 and S4). A comparable dynamic behavior for imipenem (supposedly not an AcrB substrate) was observed in our previous study [55]. Second, we found an interesting correlation between the MW of the compounds and their binding preference to previously identified sub-pockets within the DP. Namely, the groove seems to contribute to the binding of medium/high MW substrates, while smaller compounds tend to cluster within the cave and interact marginally with the AP/DP Interface. The only exception to this rule seems to be ertapenem, for which the cave and the groove contribute to a similar extent to the binding, mostly because of a significant role of PHE178 for the stabilization of this compound. Tomopenem turns out to be the compound for which the residues from groove, cave, and interface contribute less to the binding. Third, another peculiar property for the carbapenems family highlighted by the MD simulations, appears to be the ability of the drugs in the hydrophobicity screen of the DP_T_. The higher SM_H_ values found for the carbapenems in comparison to SM_L_ values (Table 2) indicate a preference for a hydrophilic complementary interactions surface between the drug and the protein, although the prevalent composition of the DP_T_ is hydrophobic.

In a broader perspective, our findings are in agreement with previous results by Takatsuka and Nikaido [44], who investigated the binding of a few dozen compounds to the DP_T_ of AcrB. Even if not explicitly mentioned by the authors, a link between the MWs of compounds and their binding preference towards different regions of the pocket is detectable from the analysis of their data. Indeed, we noticed that compounds such as doxorubicin, novobiocin, erythromycin, taurocholate, rifampicin, etc., were predicted to be groove binders, while low-MW compounds such as chloramphenicol, SDS, 1-(1-naphthylmethyl)-piperazine, etc., were predicted to be cave binders. Therefore, the present findings could represent a more general feature related to the mechanism by which AcrB achieves its polyspecificity, namely the ability to accommodate for multiple substrates not only at different pockets located in the different monomers of the transporter, but also at different sub-pockets within the same multi-functional site [42,47].

Unfortunately, we were unable to find any additional parameter correlating with the binding preferences of the carbapenems investigated in this work. However, with the limitations of the adopted computational approaches in mind (such as insufficient sampling of the possible conformations of the molecules within the DP_T_, intrinsic limitation of force fields in describing microscopic changes that could happen within the pocket upon recognition and binding, to cite a few), in combination with accurate assays to directly measure efflux of substrates [53,67,74,75,76], we believe that the computational protocol presented in this study represents a valuable source of information to experimentalist aiming at rationalizing microbiological data.

## 4. Materials and Methods

### 4.1. Molecular Docking

Following the protocol adopted in previous investigations [55,59], we first performed an extensive blind docking campaign using AutodockVINA (The Scripps Research Institute, La Jolla, CA, USA) [77] by adopting a rectangular search space of size 125 × 125 × 110Å^3^ enclosing the whole portion of the protein potentially exposed to ligands. Flexibility of both docking partners was considered indirectly, by employing ensembles of conformations. For each compound, representative conformations obtained from an explicitly solvated MD simulation of 1 µs were used (data available at www.dsf.unica.it/translocation/db) [78], see also Table S2 for a list of some molecular properties). In all cases we considered only the most populated charge/protonation state at physiological pH = 7.4 [78]. For the receptor, conformations including X-ray structures as well as derived from µs-long MD simulations were considered [42].

The choice of the initial conformations was limited to one pose within the DP_T_ for all molecules. Starting poses were selected according to the estimated binding affinity and the fraction of contacts made by each molecule within the DP_T_ (residues lining this region are listed in Table S1). We identified the docking poses within the DP_T_ as those in contact (3.5 Å cut-off distance) with at least 30% of the total number of residues lining this site. In addition, the position and orientation of the molecules were also taken into account to evaluate the effect on their behavior inside this region. The number of docking poses identified for each compound according to this criterion, together with the corresponding “average binding affinity” calculated from the scoring function of AutodockVINA [77] are reported in Table S2. Importantly, a statistical contact analysis performed for compounds co-crystallized with AcrB revealed how the adopted docking protocol was able to identify the relevant residues in contact with the ligand in each of the X-ray structures considered (data not shown). The 2D interaction patterns of the selected docking poses for each compound are shown in Figure S4.

### 4.2. MD Simulations and Binding Free-Energy Calculations

The initial coordinates of the AcrB-molecule complexes were taken from selected docking poses. Due to the large number of compounds considered, we employed a reduced model of the AcrB protein that does not contain the transmembrane domains, and consequently does not require modelling the phospholipidic membrane. This model has been validated in previous studies [36,37]. Each system was immersed in a box containing TIP3P water molecules [79] and an adequate number of K^+^ counterions, in order to neutralize the negative net charge of the system. An osmolarity of 0.15 M was reached by adding an appropriate number of K^+^/Cl^−^. The ff14SB version of the AMBER force field [80] were adopted for AcrB, while the General Amber Force-Field parameters [81] adopted for each molecule were taken from Ref. [78]. The systems were then minimized with a combination of steepest descent and conjugate gradient methods gradually releasing positional restraints applied. The AcrB/molecule complexes were heated from 0 to 310 K in two steps: a 1 ns heating from 0 to 100 K in a canonical ensemble (NVT), followed by 5 ns heating to reach 310 K in an isothermal–isobaric ensemble (NPT). Multiple equilibration steps of 500 ps each until the stabilization of the box dimensions were performed in the NPT ensemble. A Langevin thermostat using a collision frequency of 1 ps^−1^ and a Berendsen isotropic barostat [82] maintained a constant temperature, and an average pressure of 1 atm, respectively. A time step of 2 fs was used during the equilibration protocol. The 100 ns long MD simulations were carried out using the PMEMD module of AMBER 18 (University of California, San Francisco, CA, USA) [83] with a time step of 4 fs in the NVT ensemble, after application of the hydrogen mass repartitioning [84]. Coordinates were saved every 100 ps. Long-range electrostatic interactions were calculated using the particle mesh Ewald method with a cut-off of 9 Å [85]

Root mean square deviations (RMSDs) were calculated using the *cpptraj* module of AMBERTools and VMD (Beckman Institute, University of Illinois at Urbana-Champaign, USA) [86]. A cluster analysis using the *cpptraj* module of AMBERTools identified the most populated configurations sampled during the simulation with a fixed clustering radius of 3.5 Å. Only the most populated cluster were further taken into account for evaluating the free energies of binding using the molecular mechanics generalized Born surface area (MM-GBSA [87]) approach. The complementarity of the hydrophobic and hydrophilic properties of the compounds and the DP_T_ has been evaluated using PLATINUM webserver [88]. A more detailed description of the MM-GBSA method and the hydrophobic surface analysis implemented in PLATINUM can be found in the Supporting Information of Ref. [55,59]. The starting X-ray crystal configurations and the selected docking poses as well as the “final” cluster representative of each MD simulation can be visualized on-line through the NGL-viewer [89] at www.dsf.unica.it/translocation/docking.

## 5. Conclusions

In this work we combined different computational techniques to investigate the interaction of carbapenem antibiotics with the experimentally known periplasmic binding pocket DP_T_ of AcrB, the major efflux transporter from *E. coli*. Despite the acknowledged limitations, our protocol was able to identify specific residues within the DP_T_ of AcrB playing a prominent role in the binding affinity of selected members of the carbapenem family. Importantly, this study allowed the identification of subtle but relevant differences in compound/AcrB interactions, which can be hardly detected by other means (namely MIC measurements). As such, it proved to be a precious tool to rationalize molecular recognition of carbapenems by AcrB at an atomistic level. Specifically, we highlighted a correlation between the molecular weight of compounds and their binding preference to previously identified sub-pockets within the DP_T_, which could represent a general feature of the polyspecificity of AcrB. Altogether, these pieces of information could help in the design of compounds that are less affected by the activity of efflux pumps, or inhibitors able to hinder their function.

## Figures and Tables

**Figure 4:**
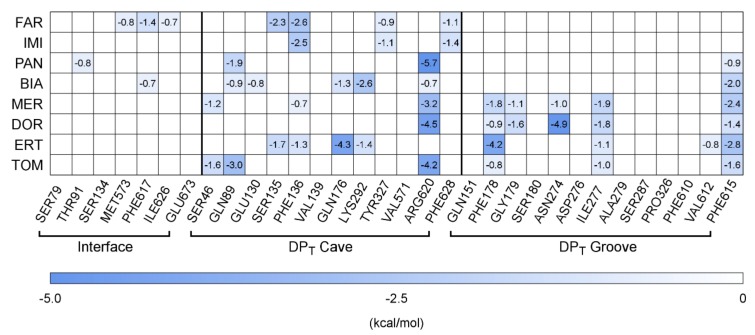
Per-residue contributions to the free-energy of binding (kcal/mol) of carbapenems to AcrB. See Figure 3 for further details.

**Table 1:** List of substrates and inhibitors co-crystallized with AcrB and considered in this study, along with their molecular identifier and molecular weight (MW). The central columns report the hydrophobic (SM_L_), and hydrophilic surface matching (SM_H_), and the percentage of water molecules in the first solvation shell (Hyd) calculated against molecular dynamics (MD) simulations of the corresponding compound in water solution. The remaining columns report respectively the free energy of binding (ΔG_b_) for the most populated cluster trajectory identified for each simulation, and the percentage contribution to ΔG_b_ from residues lining the interface between access pocket and DP_T_, the cave, and the groove regions of the DP_T_ (see Figure 1 and residues list in Table S1).

Co-Crystallized AcrB Compound	ID	MW (Da)	SM_L_	SM_H_	Hyd (%)	ΔG_b_ (kcal/mol)	Contribution to ΔG_b_ (%) Interface Cave Groove		
Rhodamine-6G	RDM	444	0.80	0.01	35 ± 8	−38.3 ± 3.3	2	5	29
Minocycline	MIN	458	0.80	0.70	34 ± 8	−29.3 ± 4.8	1	5	53
MBX3132	MBX	495	0.87	0.63	30 ± 8	−53.6 ± 4.6	2	18	24
D13-9001	P9D	693	0.96	0.91	40 ± 7	−52.3 ± 4.9	2	22	25

**Table 2:** Structural and energetic features of the carbapenem-AcrB interaction. See Table 1 for details.

Carbapenem Antibiotic	ID	MW (Da)	SM_L_	SM_H_	Hyd (%)	ΔG_b_ (kcal/mol)	Contribution to ΔG_b_ (%) Interface Cave Groove		
Faropenem	FAR	284	0.45	0.81	27 ± 9	−25.5 ± 3.5	12	31	5
Imipenem	IMI	299	0.11	0.21	37 ± 13	−25.1 ± 4.7	6	24	4
Panipenem	PAN	339	0.01	0.80	50 ± 11	−27.6 ± 5.0	4	23	5
Biapenem	BIA	350	0.04	0.60	45 ± 13	−30.5 ± 5.5	5	20	7
Meropenem	MER	383	0.29	0.73	35 ± 8	−30.8 ± 4.3	1	15	29
Doripenem	DOR	420	0.35	0.84	47 ± 10	−33.6 ± 5.5	0	14	33
Ertapenem	ERT	475	0.30	0.51	42 ± 9	−33.8 ± 7.8	3	28	31
Tomopenem	TOM	539	0.01	0.54	50 ± 9	−32.6 ± 6.7	0	15	12

## Supplementary Materials

Supplementary materials can be found at https://www.mdpi.com/1422-0067/21/3/860/s1.

## Abbreviations

AP	Access Pocket
DP	Distal Pocket
EG	Exit Gate
HP-Trap	Hydrophobic Trap
MD	Molecular Dynamics
MDR	Multi Drug Resistance
MIC	Minimum Inhibitory Concentration
MW	Molecular Weight
RMSD	Root Mean Square Displacement
RND	Resistance Nodulation and cell Division
TM	Transmembrane
