# Supplementary material for: Molecular Interactions of Carbapenem Antibiotics with the Multidrug Efflux Transporter AcrB of Escherichia coli
Source: Int J Mol Sci. 2020 Jan 29;21(3):860. doi: 10.3390/ijms21030860 (PMC7037162; doi:10.3390/ijms21030860)
Supplement: Supplementary file 1 [file ijms-21-00860-s001.pdf]

Supporting Information

Molecular interactions of carbapenem antibiotics with the multidrug efflux transporter AcrB of *Escherichia coli*

Alessio Atzori[1], Giuliano Malloci[1,*], Francesca Cardamone[1], Andrea Bosin[1], Attilio V. Vargiu[1,*] and Paolo Ruggerone[1]

[1] Department of Physics, University of Cagliari, 09042 Monserrato (CA), Italy
* Correspondence: giuliano.malloci@dsf.unica.it, vargiu@dsf.unica.it; Tel.: +39-070-675-4911 (G.M. and A.V.V.)

Supplementary Tables

Table S1 – Residue composition of AcrB regions of specific interest for this study.

Access Pocket (AP)	SER79, THR91, SER134, SER135, LYS292, MET573, MET575, GLN577, PHE617, THR624, MET662, PHE664, PHE666, ASN667, LEU668, LEU674, THR676, ASP681, ARG717, ASN719, GLU826
Deep Binding Pocket (DP)	SER46, GLN89, SER128, GLU130, SER134, PHE136, VAL139, GLN176, LEU177, PHE178, GLY179, SER180, GLU273, ASN274, ASP276, ILE277, TYR327, MET573, PHE610, VAL612, PHE615, PHE617, ARG620, PHE628
Switch Loop	GLY616, PHE617, ALA618, GLY619
Bottom Loop	ALA670 – THR676
Hydrophobic (HP) Trap	PHE136, PHE178, PHE610, PHE615, PHE628
External Cleft	ASP566, PHE664, PHE666, LEU668, GLU673, THR676, ARG717, LEU828
Exit Gate (EG)	GLN124, GLN125, TYR758, LYS770
Interface	SER79, THR91, SER134, MET573, PHE617, ILE626, GLU673
DP Cave	SER46, GLN89, GLU130, SER135, PHE136, VAL139, GLN176, LYS292, TYR327, VAL571, ARG620, PHE628
DP Groove	GLN151, PHE178, GLY179, SER180, ASN274, ASP276, ILE277, ALA279, SER287, PRO326, PHE610, VAL612, PHE615

Table S2 *Physical-chemical features of the compounds considered in study. ASA_{Hph}/ASA and ASA_{Pol}/ASA represent the fraction of hydrophobic and polar molecular surface areas. The total number of docking poses and corresponding average binding affinity obtained from the blind docking campaign performed with Autodock VINA are reported in the last two columns.*

Compound	XlogP3	Charge	ASA_{Hph} /ASA (%)	ASA_{Pol} /ASA (%)	# of poses	Binding affinity (kcal/mol)
Substrates/Inhibitors for which X-ray co-crystal structure is available						
Minocycline	1.85	0	67	33	21	-11.0 ± 0.3
Rhodamine 6G	6.55	1	87	13	34	-10.9 ± 0.5
MBX3132	3.80	0	88	12	48	-10.6 ± 0.4
D13-9001	1.94	-1	77	23	76	-13.0 ± 0.6
Carbapenems						
Faropenem	0.27	-1	70	30	13	-8.3 ± 0.3
Imipenem	-0.32	0	64	36	4	-8.5 ± 0.3
Panipenem	-2.19	0	73	27	7	-9.1 ± 0.1
Biapenem	-2.32	0	65	35	6	-9.2 ± 0.3
Meropenem	-2.36	0	71	29	13	-9.4 ± 0.5
Doripenem	-3.42	0	55	45	5	-9.8 ± 0.4
Ertapenem	-1.46	-1	58	42	35	-11.3 ± 0.3
Tomopenem	-2.87	1	74	26	10	-11.3 ± 0.5

Table S3. *Collection of the MIC values for the carbapenem antibiotics considered in this work. Data reported in the EUCAST QC Tables v.8.0 and v.9.0 [1] are marked with one and two asterisks, respectively.*

**Antibiotics**	**ID**	**MIC μg/ml**	**Method**	**Ref**
Faropenem	FAR	0.39	Agar dilution	[2]
Imipenem	IMI	0.06-0.25**	Agar dilution	[1]
Panipenem	PAN	0.12	Agar dilution	[3]
Biapenem	BIA	0.03	Agar dilution	[3]
Meropenem	MER	0.008-0.064**	Agar dilution	[1]
Doripenem	DOR	0.016-0.064*	Agar dilution	[3]
Ertapenem	ERT	0.004-0.016**	Agar dilution	[1]
Tomopenem	TOM	≤0.03	Microdilution broth method	[4]

Supplementary Figures

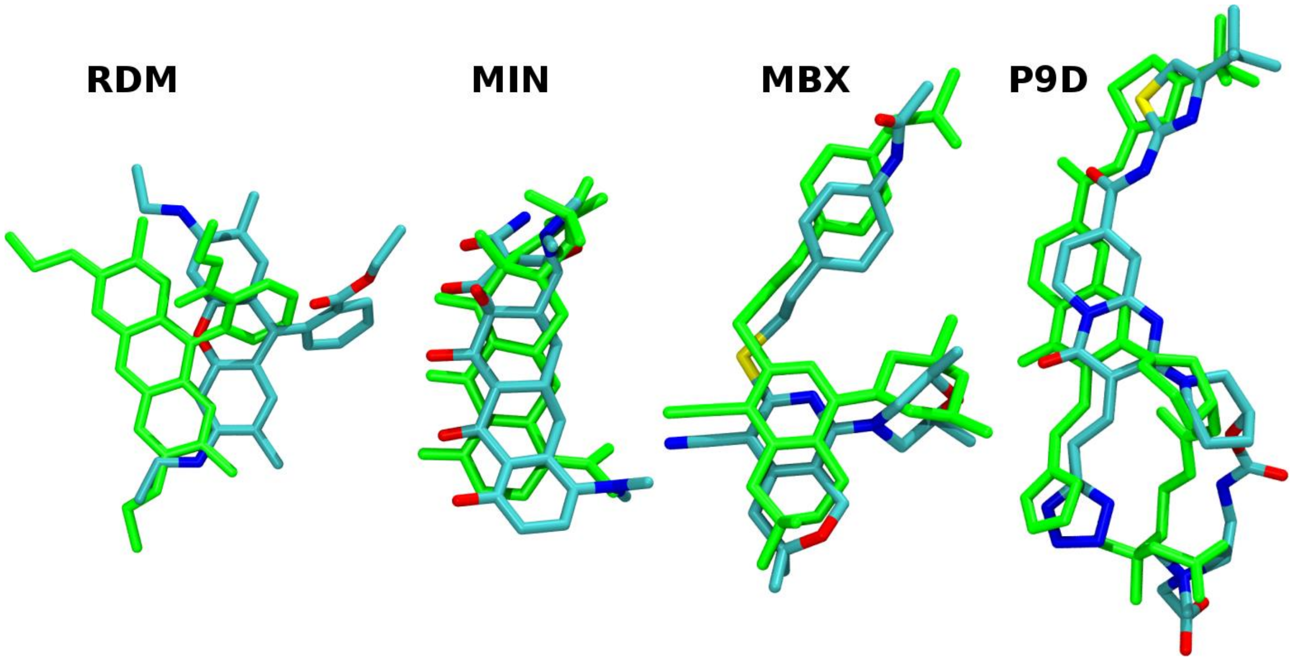

Figure S1 - *Comparison between the X-ray configuration of RDM, MIN, MBX, and P9D (green) and the cluster representative extracted from the MD simulations.*

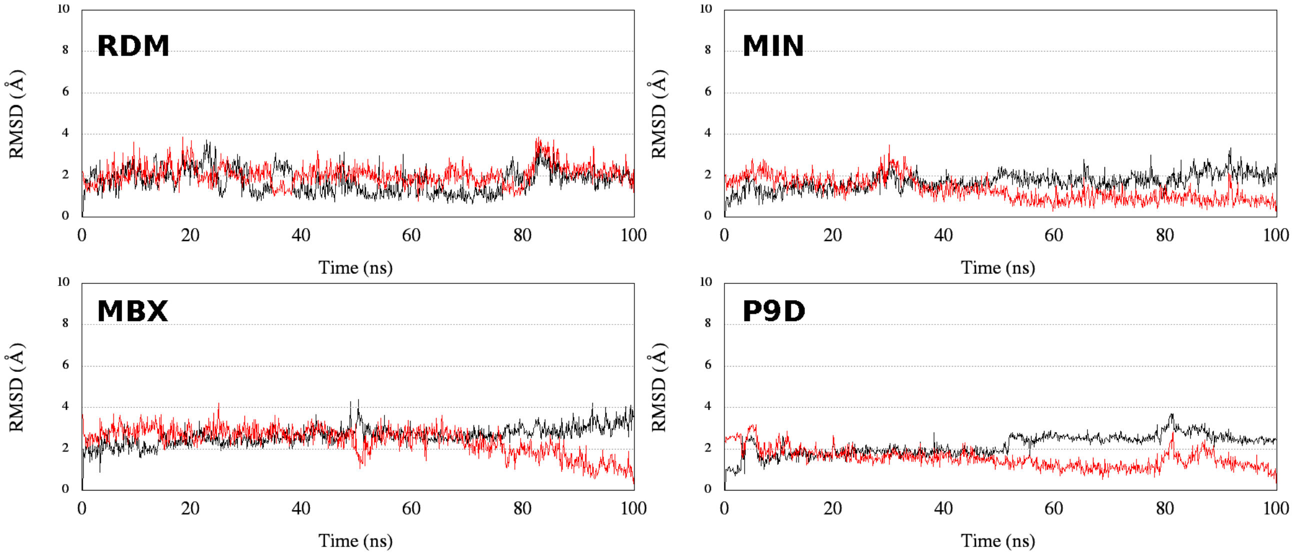

Figure S2 – *RMSD evolution during the MD simulation for the substrates and inhibitors considered. The black and red curves correspond to the RMSD computed with respect to the first and last frame of the production phase, respectively.*

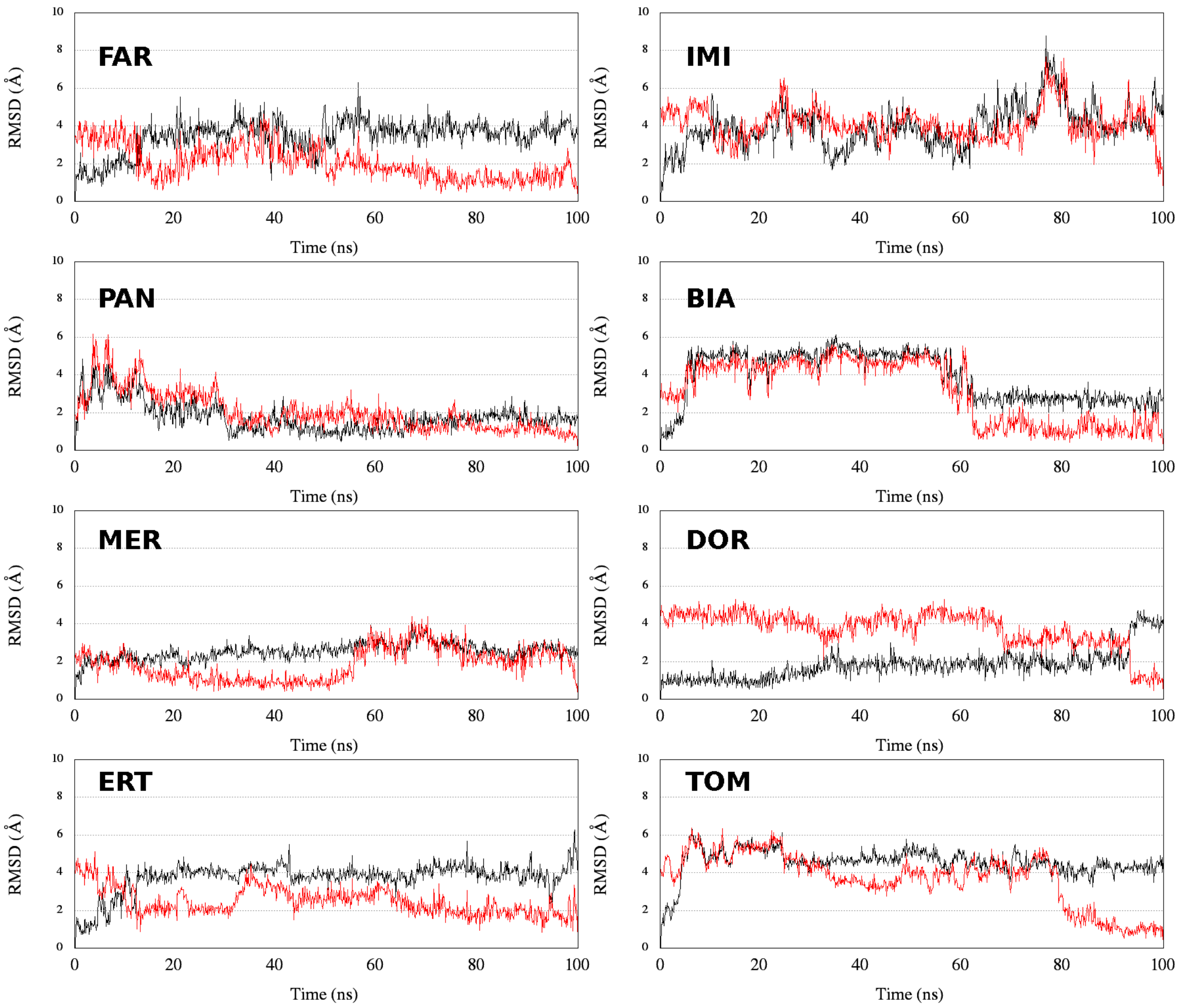

Figure S3 – RMSD evolution during the MD for the carbapenem antibiotics considered. See Figure S3 for further details.

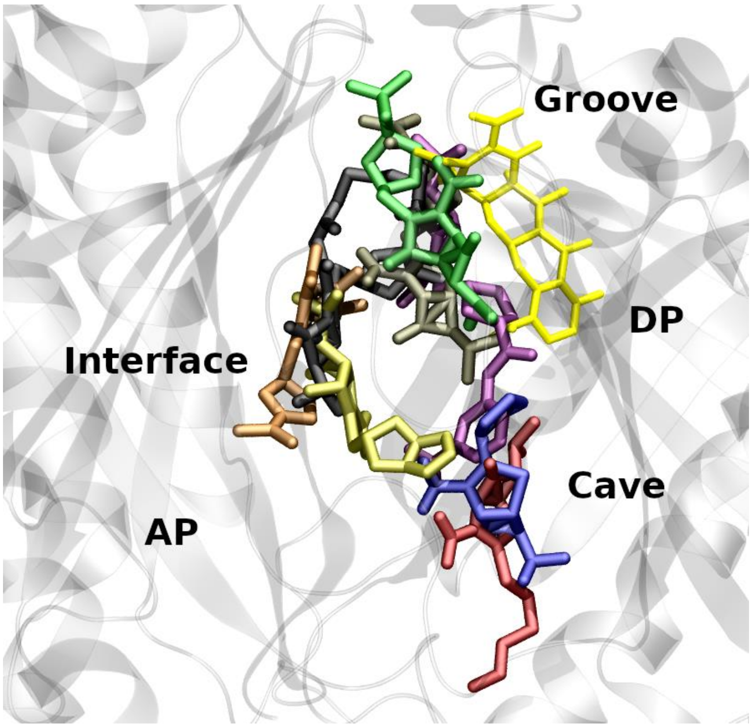

Figure S4 - Spatial distribution of the cluster representative extracted from MD simulations of FAR (blue), IMI (red), PAN (orange), BIA (yellow), MER (tan), DOR (green), ERT (purple), and TOM (black). As a term of reference, the X-ray configuration of MIN (taken from PDB_ID: 4DX5) is shown in light yellow.

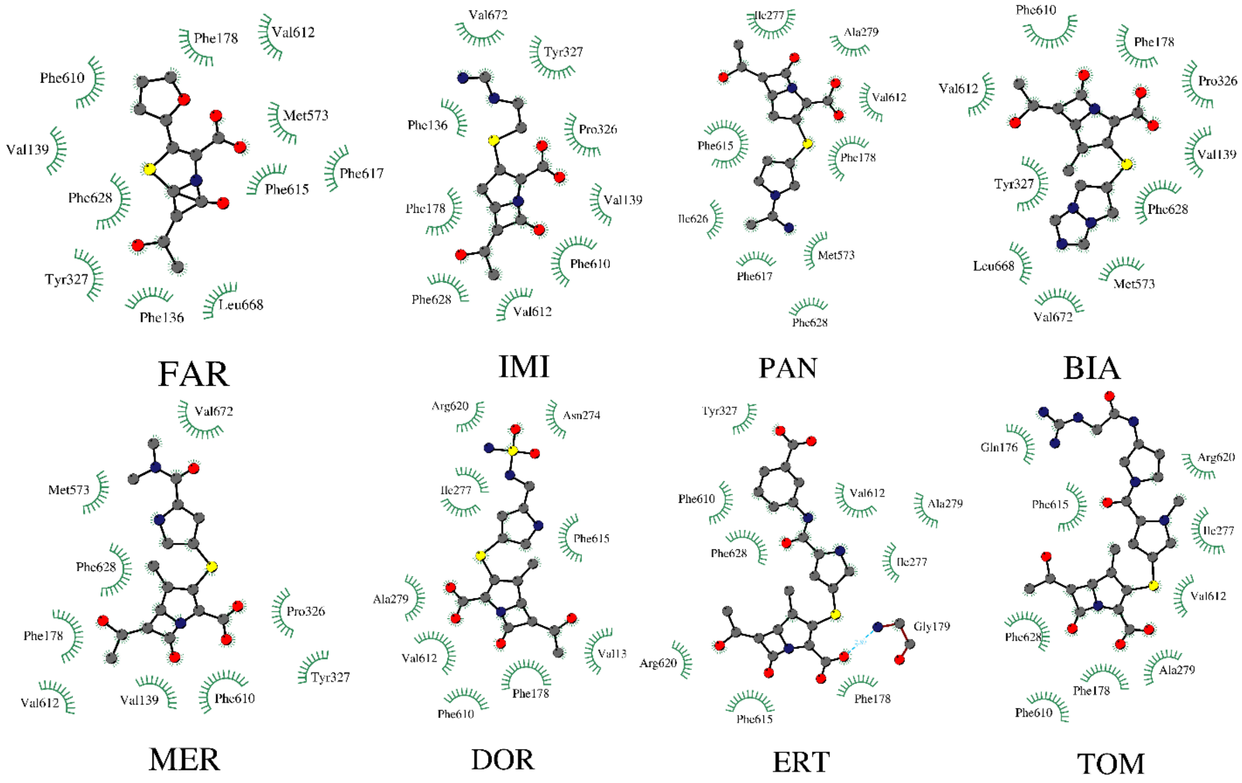

Figure S5 – 2D interaction patterns for the selected docking poses obtained for each carbapenem. The hydrophobic contacts hydrogen bonds are shown in green and light blue colors, respectively. The picture has been obtained with the LigPlot+ software [5].

Supplementary references

[1] "The European Committee on Antimicrobial Susceptibility Testing. Routine and extended internal quality control for MIC determination and disk diffusion as recommended by EUCAST. Version 9.0, 2019. http://www.eucast.org."

[2] Nishino K, Yamada J, Hirakawa H, Hirata T, Yamaguchi A. Roles of TolC-dependent multidrug transporters of Escherichia coli in resistance to β-lactams. Antimicrob Agents Chemother 2003, 47, 3030–3.

[3] Yamachika S, Sugihara C, Kamai Y, Yamashita M. Correlation between penicillin-binding protein 2 mutations and carbapenem resistance in Escherichia coli. J Med Microbiol 2013, 62, 429–36.

[4] Koga T, Abe T, Inoue H, Takenouchi T, Kitayama A, Yoshida T, Masuda N, Sugihara C, Kakuta M, Nakagawa M, Shibayama T, Matsushita Y, Hirota T, Ohya S, Utsui Y, Fukuoka T, Kuwahara S. In vitro and in vivo antibacterial activities of CS-023 (RO4908463), a novel parenteral carbapenem. *Antimicrob Agents Chemother* **2005**, *49*, 3239–50.

[5] Laskowski, R.A.; Swindells, M.B. LigPlot+: multiple ligand-protein interaction diagrams for drug discovery. *J Chem Inf Model* **2011**, *51*, 2778-2786.
